# Self-management for osteoarthritis of the knee: Does mode of delivery influence outcome?

**DOI:** 10.1186/1471-2474-11-56

**Published:** 2010-03-24

**Authors:** Sophie Coleman, Jean McQuade, Jessica Rose, Charles Inderjeeth, Graeme Carroll, N Kathryn Briffa

**Affiliations:** 1 Department of Physiotherapy, Curtin Health Innovation Research Institute, Curtin University of Technology, Bentley, Western Australia 6102, Australia; 2 Arthritis Foundation of Western Australia, PO Box 34, Wembley, Western Australia 6914, Australia; 3 Department of Geriatric Medicine and Rheumatology, North Metropolitan Area Health Service,, University of Western Australia, Verdun St, Nedlands, Western Australia 6009, Australia; 4 ArthroCare Pty Ltd, Department of Rheumatology, Fremantle Hospital, University of Notre Dame Australia, University of Western Australia, Western Australia 6050, Australia

## Abstract

**Background:**

Self-management has become increasingly popular in the management of chronic diseases. There are many different self-management models. Meta analyses of arthritis self-management have concluded that it is difficult to recommend any one program in preference to another due to inconsistencies in the study designs used to evaluate different programs.

The Stanford Arthritis Self-Management Program (ASMP), most commonly delivered by trained lay leaders, is a generic program widely used for people with rheumatological disorders. We have developed a more specific program expressly for people with osteoarthritis of the knee (OAKP). It includes information designed to be delivered by health professionals and results in improvements in pain, function and quality of life.

*Aim: *To determine whether, for people with osteoarthritis (OA) of the knee, the OAKP implemented in a primary health care setting can achieve and maintain clinically meaningful improvements in more participants than ASMP delivered in the same environment.

**Methods/Design:**

The effectiveness of the programs will be compared in a single-blind randomized study.

*Participants: *146 participants with established OA knee will be recruited. Volunteers with coexistent inflammatory joint disease or serious co-morbidities will be excluded.

*Interventions*: Participants will be randomised into either OAKP or ASMP groups and followed for 6 months.

*Measurements*: Assessments will be immediately before and after the intervention and at 6 months. Primary outcome measures will be WOMAC and SF-36 questionnaires and a VAS for pain. Secondary outcomes will include balance, tested using a timed single leg balance test and a timed step test and self-efficacy. Data will be analysed using repeated measures ANOVA.

**Discussion:**

With an aging population the health care costs for people with arthritis are ever increasing. Although cost analysis is beyond the scope of this study, it is reasonable to expect that costs will be greater when health professionals deliver self-management programs as opposed to lay leaders. Consequently it is critical to examine the relative effectiveness of the primary care management strategies available for OA.

**Trial Registration:**

This study is registered with the Australian New Zealand Clinical Trials Registry: 12607000031460

## Background

Chronic disease is a major concern with an ageing population, and arthritis is one of the most prevalent chronic diseases affecting 16.7% of the population in Australia [1]. Osteoarthritis is the most common form of arthritis affecting 25% of the population over the age of 65 years. The joint most frequently affected is the knee [2].

Self-management interventions are becoming increasingly popular for many chronic diseases, however the length of program and mode of delivery varies greatly between programs and between illnesses [3]. The programs may utilise a combination of health professionals (physiotherapists, occupational therapists, nurses and dieticians), trained health educators, mental health workers, and occasionally physicians, for program delivery. Other programs have lay leaders who usually deliver a scripted program. The mode of delivery also varies greatly from face to face to audiotape; group or individual contact and more recently internet based programs [4].

Comparing different self-management models is difficult as the needs of people with chronic diseases differ according to their illness. Asthma and diabetes self-management programs emphasise medication delivery and compliance, whereas self-management for other conditions may focus more on pain management strategies. Even within the broad category of arthritis, the self-management needs of people with different types of arthritis such as rheumatoid arthritis or osteoarthritis are not the same.

One widely utilised arthritis self-management program is the Stanford University's Arthritis Self Management Progam (ASMP), which is delivered by trained lay leaders [5]. The ASMP has been tested widely. The majority of studies have been conducted in the USA or UK. Many, but not all of these studies report program efficacy. Systematic reviews of self-management interventions that include the ASMP have shown there is a trend towards a small benefit for people with arthritis, but the results were not statistically significant and there was a suggestion of publication bias [3,6,7]. At this stage, it is not possible to unequivically claim that ASMP is effective.

In view of the high prevalence of osteoarthritis of the knee and the absence of unequivocal evidence of effectiveness of ASMP, we developed an education self-management program specifically for people with osteoarthritis of the knee (OAKP). The program is delivered by health professionals with information and instruction that utilises their knowledge and skills within a self-management construct. The OAKP has been tested in a quality assurance project [8] and a randomised controlled trial (RCT) the results of which show improvements in quality of life, pain and function compared to a control group [9].

With an aging population the costs associated with arthritis are ever increasing [1]. Although cost analysis is beyond the scope of this study, it is reasonable to expect that costs will be greater when health professionals deliver self-management programs as opposed to lay leaders. Therefore it is important to establish whether delivery by health professionals results in added benefits and/or improvements in outcomes. At present both ASMP and OAKP are offered as clinical services at Arthritis WA.

Accordingly, we propose to conduct a RCT to examine the differences between OAKP, directed and delivered by health professionals, and ASMP, delivered by trained lay leaders.

### Aims

To compare the effectiveness of the OAKP and ASMP for people with OA knee.

### Hypothesis

A greater proportion of people with osteoarthritis of the knee that complete the OAKP will report clinically meaningful improvements in pain, knee function and quality of life, at 8 weeks, and 6 months compared to those who complete the ASMP.

## Methods/Design

### Study Design

**Figure 1:**
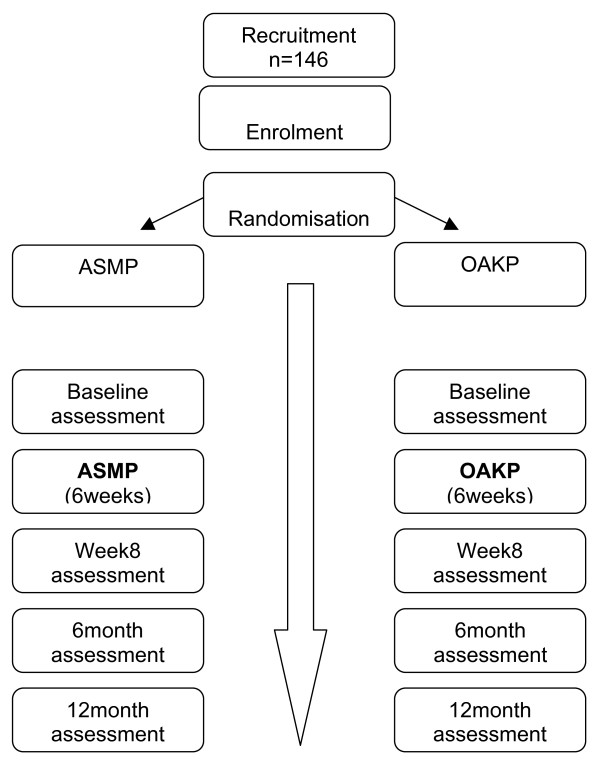
**Study Design Flow Chart**.

A two-group single blind, randomised, repeated measure study design will be used to compare the programs (Figure 1). Participants in both groups will continue to receive standard medical management as required.

### Group allocation

To ensure manageable numbers for intervention groups, participants will be randomised in blocks. Pre-prepared cards indicating group assignment will be placed in sealed opaque envelopes and drawn as a lottery by a third party for allocation to treatment groups. Allocation will not take place until a whole block has been recruited in order to ensure optimum group sizes. This method of randomisation worked successfully for a previous OAKP RCT [10].

### Subjects

As this study will evaluate a clinical service currently provided by Arthritis WA, the study recruitment strategies and selection criteria have been selected to operate within that context. The participants will be men and women with established OA of one or both knees. The operational definition for OA knee used for the OAKP is OA of the knee diagnosed either by clinical examination or by radiological (X-Ray) evidence. The participant will be required to have a referral and a definitive diagnosis of OA made by a family physician/general medical practitioner or specialist physician in order to be eligible to participate in the study. In addition, the American Rheumatology Association clinical classification algorithm will be applied. The overall sensitivity and specificity of this method are 89% and 88% respectively [11]. The inclusion criteria for determining suitability have previously been used for a quality assurance study and a randomised RCT [8,10] and in both it was found to work well. Participants will not be excluded on the basis of severity of symptoms. They will be required to be ≥ 18 years of age, English speaking and to provide consent to randomisation as demonstrated by signed written authority. Participants with rheumatoid arthritis or other inflammatory joint disease, serious co-morbidities, those who plan to have knee surgery within 6 months of commencing the study or who have physical impairments that preclude fulfilment of the requirements of either program will be excluded (Additional file 1).

Recruitment strategies will include active promotion to general practitioners, rheumatologists and health professionals through professional societies. The study will also be offered to those people with osteoarthritis of the knee who make general inquiries to Arthritis WA, and to the general public through advertising and media coverage.

#### Interventions

Both programs will be delivered over a six-week period with participants attending one session of two and a half hours per week. To facilitate optimum group dynamics, the target size for each group will be 12 participants, although this may vary as a result of recruitment and randomisation.

There will be a pool of 4 group leaders for each program, health professionals for OAKP and lay leaders for ASMP. The health professionals will be trained in delivery of self-management programs and the lay leaders will be experienced ASMP facilitators who have completed the "Train the Trainer course" conducted at Arthritis WA, under licence from Stanford University. Facilitators will work in pairs when delivering the programs.

The fidelity of OAKP and ASMP will be maintained as both programs have manuals for course delivery.

##### OAKP

The health professionals delivering the OAKP will be required to have the knowledge and skills to present information on disease specific topics, exercise advice, and to accurately respond to complex questions.

The Program includes the following aspects of care:

• Osteoarthritis - explanation and implications

• Pain management strategies (cognitive and pharmacological)

• Fitness and exercise (strength, flexibility, aerobic and balance)

• Joint protection

• Nutrition and weight control

• Medication (type, interactions, current trends)

• Correct use of analgesia (use of, therapeutic dosing, types of analgesia, side effects)

• Balance, proprioception and falls prevention,

• Coping with negative emotions

• Self-management skills (SMART goals [**S**pecific, **M**easurable, **A**chievable, **R**ealistic, **T**ime-framed], problem solving, modelling, positive thinking, improving self-efficacy).

##### ASMP

As the ASMP was designed to be delivered by lay leaders to people with a variety of musculoskeletal conditions such as rheumatoid arthritis, osteoarthritis, fibromyalgia and systemic lupus erythematosus, the course content is more generic.

Subjects covered in ASMP include [12]:

• Techniques to deal with problems such as pain, fatigue, frustration and isolation,

• Appropriate exercise for maintaining and improving strength, flexibility, and endurance,

• Appropriate use of medications,

• Communicating effectively with family, friends and health professionals,

• Healthy eating,

• Making informed treatment decisions,

• Disease related problem solving

• Getting a good night's sleep.

### Ethical Issues

This study has been approved by the Human Research Ethics Committee at Curtin University of Technology and meets with CONSORT guidelines. It is registered with the Australian New Zealand Clinical Trials Registry, number: 12607000031460.

All participants will provide written informed consent prior to randomisation. Data access and storage will be in keeping with National Health and Medical Research Council guidelines. License agreements have been obtained for SF-36 and WOMAC Questionnaires.

### Instruments & Assessments

#### Baseline Screening/Assessment

Telephone screening will be conducted with people who enquire to enrol into the study. Suitable candidates will have study information sent to them. Following enrolment and written consent, participants will be randomised into groups. At the baseline assessment, demographic information will be collected including: past medical history, current medications including prescribed, over the counter and natural therapies. Records of medical practitioner referral, diagnosis and X-Ray reports will be collected.

#### Response to Intervention

Participants will be assessed using the following outcome measures at baseline (Week 1), immediately post-intervention (Week 8), and at 6 months after commencing the program. In addition, VAS pain will be assessed on a week-to-week basis during the first 8 weeks- that is the two assessment weeks and the 6 intervention weeks. A research assistant who will remain blind to group allocation will perform all assessments at all time-points. The primary outcomes for the study will be health status, quality of life and pain severity.

### Outcome measures

#### Western Ontario and McMaster Universities (WOMAC) Osteoarthritis index

WOMAC measures health status and assesses pain, stiffness and physical function in patients with OA of the hip or knee. For the purpose of this study the Likert (WOMAC LK3.0) format will be used. The WOMAC questionnaire is self-administered and can be completed in less than 10 minutes. Two major validity studies have shown WOMAC pain, stiffness and physical function subscales are valid and that the questionnaire is reliable and sensitive enough to detect changes in health status following a variety of interventions [13,14].

#### The Short Form 36 Questionnaire (SF-36)

The SF-36 measures quality of life and has 8 sub-components reflecting both physical and mental status. It is comprised of 36 questions, is self-administered and can be completed in approximately 15 minutes. All estimates of score reliability, from 14 separate studies, for each of the 8 sub-categories of the SF-36 exceed accepted standards for measures used in group comparisons [15]. The SF-36 has been extensively validated in many English speaking countries of the world including Australia [16]. The rationale for using both WOMAC and the SF-36 for this study is that a combined approach using both generic and knee specific measures is considered likely to prove best for knee related problems [17].

#### Visual Analog Scale (VAS)

VAS will be used to measure participants' pain severity. The VAS is a single dimension horizontal scale, which consists of a 10 cm line on which participants rate their pain from 0-10. Participants are required to place a vertical pen line through the scale at the level of their pain- 0 reflecting No Pain and 10 reflecting the Worst Pain imaginable. The VAS is well established in clinical practice for measuring pain post -surgery, following drug therapy and in response to other interventions in arthritis populations [18].

#### Modified Timed Up and Go Test

This test is a modification of the "Timed Up and Go" test (mTUG), used to assess basic functional mobility in the elderly [19]. TUG is a widely used clinical outcome tool in which the time taken to stand from sitting, walk 3 m, turn around, return to the chair and sit down is measured. This test is reliable and valid [19]. In this study, the time taken to ascend and descend a 15 cm step has been added to the outward walk, and the length of the walk has been extended to 4 m.

#### Step Test

This is a test of dynamic standing balance. It involves stepping one foot on, then off a block as quickly as possible in a set time period of 30 seconds. It has high test-retest reliability (ICC > 0.90) good concurrent validity and is sensitive to changes in performance over time [20].

#### Timed Single Leg balance Test

This is a simple test that assesses the difficulty a person has standing on one leg. The score is the total time (in seconds, to a maximum of 30 seconds) standing on one leg. It is thought that this test is a good predictor of falls in the elderly [21] and is reliable and valid (rc = 0.69) [22].

#### Arthritis Self-Efficacy Questionnaire

This is an 8-item scale. Participants are asked to complete the questionnaire by circling the number that best reflects the degree to which they are confident they can complete a described task at the present time. The score is the total of the 8 items. This questionnaire has been widely used in arthritis research and has internal consistency and reliability of 0.94 [23].

### Statistical Power

Power calculations are based on the achievement of a minimal clinically meaningful improvement [24]. With an alpha (1-tailed) of 0.05 and a sample size of 73 people in each group, this study will have power of 80% to show that the response rate for ASMP is at least as high as the response rate for OAKP. This assumes that the response rates for the ASMP and OAKP groups are equal (at 38.0%, the level of response achieved for the WOMAC function scale in our earlier RCT [10]), and that a difference of 20.0 points or less is unimportant and allows for 20% drop out.

### Data Analysis

Data will be analysed in a blinded manner using SPSS v17 for Macintosh. Treatment groups will be examined for comparability at baseline using unpaired t-tests or Chi-squared test as appropriate. Main comparisons between treatment groups will be performed using an intention to treat analysis. For all subjects who complete the 6-month measurements, previous values will be carried forward to replace any interim missing values.

The proportion of participants achieving minimal clinically meaningful improvement in the health status, quality of life and pain will be computed for each group at each observation time. The effect of the treatment, in terms of the proportion showing minimal clinically meaningful improvement will be examined using Chi-square test. Separate analysis will be conducted for each of the outcome variables of interest.

Further, participants will be classified as overall responders or non-responders. A favourable response to treatment (responder) will be defined according to scenario D of the OMERACT-OARSI criteria [25]. That is, an improvement of ≥ 50% in pain or function and an absolute change of ≥ 20 points on a 100 point scale, OR an improvement of at least 2 of the following: An improvement of ≥ 20% and an absolute change of ≥ 10 in two of pain, function and global health. The proportion of participants achieving responder criteria will be computed for each group at each observation time. The effect of the treatment, in terms of the proportion of responders will be examined using Chi-square test.

Finally, repeated measures ANOVA will be used to examine the differences between groups over time. Statistical significance will be inferred at a 1-tailed p < 0.05. Results will not be adjusted for multiple comparisons as all outcomes of interest have been nominated a priori and such adjustment would likely render all findings of interest non-significant, despite their clinical importance [26]. Separate analysis will be conducted for each of the outcome variables of interest.

## Discussion

Meta-analyses of self-management have all concluded that it is difficult to compare models between different chronic conditions, and this is also the case with different types of arthritis [3,7,27]. Many disease states exist under the banner of arthritis, and all of them have different symptoms and requirements. People with any type of arthritis can enrol in ASMP, as it is a generic program. Facilitation by lay leaders and the variety of arthritic conditions that can be accommodated in group sessions have advantages in terms of cost and feasibility, for example in small communities.

In contrast, the OAKP is a disease specific education self-management program that was designed for facilitation by health professionals to enable more detailed information specific to OA knee to be included.

The study described in this paper will determine comparative efficacy of these programs and the results will assist in planning future arthritis self-management strategies. The widely used valid and reliable outcome measures along with design features such as randomisation and blinding will minimise bias and facilitate comparison with other studies.

## Abbreviations

ASMP: Arthritis Self-Management Program; OAKP: Osteoarthritis of the Knee Program; OA: Osteoarthritis; RCT: Randomised controlled trial; WOMAC: Western Ontario and McMaster Universities Osteoarthritis index; SF-36: Short Form 36 Questionnaire; VAS: Visual analog score; mTUG: Modified Timed Up and Go test.

## Competing interests

The authors declare that they have no competing interests.

## Authors' contributions

SC and KB were responsible for writing the study protocol and drafting the manuscript. JM, JR, CI, and GC, assisted with study design and provided comments on the drafts and all authors approved the final version of the manuscript.

## Pre-publication history

The pre-publication history for this paper can be accessed here:

http://www.biomedcentral.com/1471-2474/11/56/prepub

## Supplementary Material

Additional file 1 Eligibility Criteria Click here for file

## References

[B1] Access Economics Painful Realities: The economic impact of arthritis in Australia in 2007 2007 Canberra: Arthritis Australia

[B2] Felson D Developments in the clinical understanding of osteoarthritis Arthritis Research & Therapy 2009 11 203 10.1186/ar2531 PMC2688214 19232065

[B3] Warsi A Wang PS LaValley MP Avorn J Solomon DH Self-management education programs in chronic disease. A systematic review and methodological critique of the literature Archives of Internal Medicine 2004 164 15 1641 1649 10.1001/archinte.164.15.1641 15302634

[B4] Warsi A LaValley M Wang P Avorn J Solomon D Arthritis Self-Management Education Programs. A Meta-Analysis of the Effect on Pain and Disability Arthritis & Rheumatism 2003 48 8 2207 2213 10.1002/art.11210 12905474

[B5] Lorig K Mazonson P Holman H Evidence suggesting that health education for self-management in patients with chronic arthritis has sustained health benefits while reducing health care costs Arthritis and Rheumatism 1993 36 4 493 496 10.1002/art.1780360403 8457219

[B6] Newman S Steed L Mulligan K Self-management interventions for chronic illness The Lancet 2004 364 9444 1523 1538 10.1016/S0140-6736(04)17277-2 15500899

[B7] Weingarten SR Henning JM Badamgarav E Knight K Hasselblad V Gano AJ Ofman JJ Interventions used in disease management programmes for patients with chronic illness - which ones work? Meta-analysis of published reports BMJ 2002 325 1 8 10.1136/bmj.325.7370.925 12399340 PMC130055

[B8] Coleman S Briffa K Conroy H Prince R Carroll G McQuade J Short and medium-term effects of an education self-management program for individuals with osteoarthritis of the knee, designed and delivered by health professionals: a quality assurance study BMC Musculoskeletal Disorders 2008 9 117 10.1186/1471-2474-9-117 PMC2538526 18778467

[B9] Coleman S Conroy H Rowley E Fabling M Briffa K Carroll G Prince R McQuade J Long term effects of education in the primary health care setting using behaviour modification, and specific exercises in patients with osteoarthritis of the knee: reduced pain, improved mobility and improved quality of life American College of Rheumatology Annual Scientific Meeting: November 2002; New Orleans, USA 2002

[B10] Coleman S Briffa NK Carroll G Inderjeeth C Cook N McQuade J Effects of self-management, education and specific exercises, delivered by health professionals, in patients with osteoarthritis of the knee BMC Musculoskeletal Disorders 2008 9 133 10.1186/1471-2474-9-133 18831745 PMC2565676

[B11] Altman R Asch E Bloch D Bole G Borenstein D Brandt K Christy W Cooke T Greenwald R Hochberg M Howell D Kaplan D Koopman W Longley S Mankin H McShane D Medsger TJ Meenan R Mikkelsen W Moskowitz R Murphy W Trothschild B Segal M Sokoloff L Wolfe F Development of criteria for the classification and reporting of osteoarthritis: classification of osteoarthritis of the knee Arthritis and Rheumatism 1986 29 8 1039 1049 10.1002/art.1780290816 3741515

[B12] Arthritis Self-Management (Self-Help) Program http://patienteducation.stanford.edu/programs/asmp.html

[B13] Bellamy N WOMAC osteoarthritis index: User guide 2002 Queensland: CONROD, The University of Queensland

[B14] Roos EM Klässbo K Lohmander LS WOMAC Osteoarthritis Index: Reliability, validity, and responsiveness in patients with arthroscopically assessed osteoarthritis Scandinavian Journal of Rheumatology 1999 28 4 210 10.1080/03009749950155562 10503556

[B15] Ware JE Jr Kosinski MA Gandek B SF-36 Health Survey Manual & Interpretation Guide 2002 Lincoln RI: QualityMetric Inc

[B16] Kantz ME Harris WJ Levitsky K Ware JE Jr Davies AR Methods for assessing condition specific and generic functional status outcomes after total knee replacement Medical Care 1992 30 5 240 252 10.1097/00005650-199205001-00024 1583936

[B17] Brazier JE Harper R Munro J Walters SJ Snaith ML Generic and condition-specific outcome measures for people with osteoarthritis of the knee Rheumatology (Oxford) 1999 38 9 870 877 10.1093/rheumatology/38.9.870 10515649

[B18] Creamer P Lethbridge-Cejku M Hochberg MC Determinants of pain severity in knee osteoarthritis: effect of demographic and psychosocial variables using 3 pain measures Journal of Rheumatology 1999 26 8 1785 1792 10451078

[B19] Podsiadlo D Richardson S The timed "Up and Go": a test of basic functional mobility for frail elderly persons Journal of American Geriatric Society 1991 39 2 142 148 10.1111/j.1532-5415.1991.tb01616.x 1991946

[B20] Hill K A new test of dynamic standing balance for stroke patients: reliability, validity and comparison with healthy elderly Physiotherapy Canada 1996 48 4 257 262 10.3138/ptc.48.4.257

[B21] Vellas B Wayne S Romero L Baumgartner R Rubenstein L Garry P One-legged balance is an important predictor of injurious falls in older persons Journal of American Geriatric Society 1997 45 735 738 10.1111/j.1532-5415.1997.tb01479.x 9180669

[B22] Curb J Ceria-Ulep C Rodriguez B Grove J Guralnik J Willcox B Donlon T Masaki M Chen R Performance-based measures of function for high-function populations Journal of American Geriatric Society 2006 54 737 742 10.1111/j.1532-5415.2006.00700.x 16696737

[B23] Barlow J Williams B Wright C The reliability and validity of the arthritis self-efficacy scale in a UK context Psychology Health & Medicine 1997 2 1 3 17 10.1080/13548509708400556

[B24] Tubach F Ravaud P Baron G Falissard B Logeart I Bellamy N Bombardier C Felson D Hochberg M Heijde D van der Dougados M Evaluation of clinically relevant states in patient reported outcomes in knee and hip osteoarthritis: the patient acceptable symptom state Annals of Rheumatic Disease 2005 64 43 47 10.1136/ard.2004.023028 PMC1755171 15130902

[B25] Pham T Heijde D van der Altman R J A Bellamy N Hochberg M Simon L Strand V Woodworth T Dougados M OMERACT-OARSI Initiative: Osteoarthritis Research Society International set of responder criteria for osteoarthritis clinical trials revisited Osteoarthritis and Cartilage 2004 12 5 389 399 10.1016/j.joca.2004.02.001 15094138

[B26] Perneger T What's wrong with Bonferroni adjustments British Medical Journal 1998 316 1236 9553006 10.1136/bmj.316.7139.1236 PMC1112991

[B27] Chodosh J Morton S Mojica W Maglione M Suttorp M Hilton L Rhodes S Shekelle P Meta-analysis: chronic disease self-management programs for older adults Annals of Internal Medicine 2005 143 427 438 16172441 10.7326/0003-4819-143-6-200509200-00007

