# Supplementary material for: Self-management for osteoarthritis of the knee: Does mode of delivery influence outcome?
Source: BMC Musculoskelet Disord. 2010 Mar 24;11:56. doi: 10.1186/1471-2474-11-56 (PMC2850876; doi:10.1186/1471-2474-11-56)
Supplement: Additional file 1 — Eligibility Criteria [file 1471-2474-11-56-S1.PDF]

Inclusion	Exclusion
Confirmed OA knee	Co-existing inflammatory disease
>18 years of age	Unable to meet study time-points
English speaking	Scheduled knee replacement < 6months
Agrees to randomisation	Serious co-morbidity
Referral from physician	
Able to meet program requirements	
